# The definition of asthma remission in children: A scoping review by the WAO Paediatric Asthma Committee^☆^

**DOI:** 10.1016/j.waojou.2025.101166

**Published:** 2026-01-05

**Authors:** Eleni Anastasiou, Michael Miligkos, Yuichi Adachi, Ignacio J. Ansotegui, Héctor A. Badellino, Spiros Bekiaris, Zeinab A. El-Sayed, Adnan Custovic, Ivana Filipovic, James E. Gern, Rene Maximiliano Gómez, Cesar Pozo Beltrán, Rasha El-Owaidy, Elham Hossny, Ömer Kalayci, Peter N. Le Souëf, Mário Morais-Almeida, Antonio Nieto-Garcia, Paulo M. Pitrez, Cristina Rivas-Juesas, Alvaro Teijeiro, Wanda Phipatanakul, Jiu-Yao Wang, Gary Wong, Paraskevi Xepapadaki, Su Boon Yong, DongKeon Yon, Nikolaos G. Papadopoulos

**Affiliations:** a Allergy Department, 2nd Paediatric Clinic, National and Kapodistrian University of Athens, Athens, Greece; b Pediatric Allergy Center, Toyama Red Cross Hospital, Toyama, Japan; c Hospital Quironsalud Bizkaia, Erandio, Bilbao, Spain; d Faculty of Psychology, UCES University, San Francisco, Argentina; e Pediatric Allergy, Immunology, and Rheumatology Unit, Children's Hospital, Ain Shams University, Cairo, Egypt; f National Heart and Lung Institute, Imperial College London, London, UK; g University Hospital Dr Dragisa Misovic, Belgrade, Serbia; h Department of Pediatrics, School of Medicine and Public Health, University of Wisconsin, Madison, Wisconsin, USA; i Faculty of Health Sciences, Catholic University of Salta, Argentina; j Pediatric Allergy and Immunology, Hospital Infantil de México Federico Gómez, Mexico City, Mexico; k Hacettepe University School of Medicine, Ankara, Turkey; l School of Medicine, University of Western Australia and Telethon Kids Institute, Perth, Western Australia, Australia; m Allergy Center Hospital CUF-Descobertas, Lisbon, Portugal; n Pediatric Pulmonology and Allergy Unit, Hospital Universitari i Politècnic La Fe, Health Research Institute La Fe, Valencia, Spain; o Hospital Santa Casa de Porto Alegre, Porto Alegre, Brazil; p Department of Pediatrics, Hospital de Sagunto, Valencia, Spain; q Respiratory Department, Pediatric Hospital, Córdoba, Argentina; r Division of Allergy and Immunology, Boston Children's Hospital, Harvard Medical School, Boston, MA, USA; s Allergy, Immunology and Microbiome Research Center, China Medical University Children's Hospital, Taichung, Taiwan; t Department of Paediatrics, The Chinese University of Hong Kong, Prince of Wales Hospital, Hong Kong; u Department of Allergy and Immunology, China Medical University Children Hospital, Taichung, Taiwan; v Department of Pediatrics, Kyung Hee University College of Medicine, Seoul, Republic of Korea; w Division of Infection, Immunity and Respiratory Medicine, University of Manchester, Manchester, UK

## Abstract

**Background:**

Asthma remission has emerged as a potential therapeutic goal. However, definitions of remission have primarily focused on adult populations, with limited consensus on how remission should be defined in children.

**Objective:**

To comprehensively review how asthma remission has been defined in children and to evaluate consistency and applicability of these definitions.

**Methods:**

This scoping review was conducted following PRISMA-ScR guidelines. PubMed MEDLINE was searched for studies published between January 2010 and February 2024. Eligible studies included children with asthma and reported definitions of remission. Key remission criteria were extracted and categorized, and hierarchical cluster analysis was used to identify key patterns.

**Results:**

Twenty-nine studies met the inclusion criteria. Most (79.3%) defined paediatric asthma remission based on the absence of clinical symptoms. The most common remission timeframe ranged from 1 to 2 years. A medication-free criterion was used in 68.9% of studies. On-treatment remission was reported in the minority of studies, but it is increasingly acknowledged as a valid outcome. Objective assessments, such as normal lung function (21%) and absence of bronchial hyperresponsiveness (10.3%), were infrequently included. Cluster analysis revealed 3 main patterns for remission definition: symptom-based, event-based, and 1 including objective criteria.

**Conclusion:**

Current definitions of asthma remission in paediatric populations are predominantly symptom-based, with limited inclusion of objective physiological measures. Establishing consensus-based definitions for remission tailored to paediatric populations is essential to ensure clinical relevance and alignment with real-world disease patterns.

## Introduction

### Background

Asthma is the most common non-communicable respiratory condition in children, with its global burden still increasing in some age groups.^1^^,^^2^ It is a complex, heterogeneous and multi-trajectory disorder characterised by symptoms such as wheezing, shortness of breath and chest tightness.^3^, ^4^, ^5^ Pathological changes and physiological consequences in the airways can be observed from the early stages of the disease,^6^ particularly in school-aged children with severe asthma^7^ and preschool children with recurrent severe wheezing.^8^ However, it is unclear why some children become unexpectedly symptom-free^9^ either with or without the resolution of underlying airway abnormalities.^10^

Lexicologically, the term “remission” is defined as a temporary or permanent decrease or subsiding of the symptoms of a disease.^11^^,^^12^ Asthma often present with cyclical or intermittent symptom pattern and with pathophysiology that may include underlying subclinical inflammation and airway obstruction. In this context, in addition to the clinical absence of symptoms, remission may perceivably include biomarkers of disease processes obtained through laboratory (in vivo and in vitro) and/or lung function tests.^13^^,^^14^ Beyond the spontaneous resolution of symptoms and normalization of biomarkers, asthma remission, particularly in severe cases, may also be achieved through optimal treatment strategies, including the use of add-on biologic therapies. This concept has been established in other chronic non-communicable diseases, such as rheumatoid arthritis, where remission under treatment has become a realistic and pragmatic therapeutic goal, with disease modifying potential.^13^^,^^15^ It is unclear whether asthma biologics are disease modifying.^16^ Nonetheless, the concept of “asthma clinical remission on-treatment” is gaining attention as a potential target in the long-term management of paediatric asthma^16^ and has been introduced as a general treatment goal in several asthma management recommendations. Importantly, this concept is increasingly associated with achieving disease control through minimal therapeutic intervention, which may include the use of targeted biologic therapies, but explicitly excludes reliance on oral corticosteroids (OCS),^17^ as OCS represents non-specific suppression of symptoms rather than resolution or modification of the underlying disease process.^18^

Despite its increasing recognition as an important treatment goal for the benefit of the patients, particularly those with severe disease,^18^ there is no universally agreed-upon definition of what exactly constitutes remission in children. This lack of consensus may lead to inconsistencies in how remission is assessed and measured across studies, making it difficult to compare research findings, as well as using it in clinical practice.

Paediatric asthma poses distinct challenges, and a “one-size-fits-all” approach fails to account for the heterogeneous phenotypes and diverse disease trajectories observed in this population.^19^ In children, asthma often follows a dynamic and variable course, with some exhibiting transient wheezing that resolves with age, while others progress to persistent or severe disease. This variability is especially pronounced in children under 6 years of age, whose immune and respiratory systems are still maturing.^20^ This scoping review aims to map the available evidence on how paediatric asthma remission has been defined in the literature to date, before the remission criteria derived from adult asthma studies (such as introduction of the concept of remission on-treatment) with focus on biologic therapies are adopted in this age group.^21^^,^^22^ A scoping review was chosen because paediatric asthma remission is a broad and evolving concept, with diverse study designs and definitions that cannot be straightforwardly combined in a quantitative analysis. Conducted by the Paediatric Asthma Committee of the World Allergy Organization (WAO), this work seeks to describe the range and scope of the various definitions and initiate a structured ontology to guide future research and clinical practice.

## Methods-scoping review approach

### Protocol registration

This review was conducted as a scoping review. The reporting follows the PRISMA-ScR (Preferred Reporting Items for Systematic Reviews and Meta-Analyses Extension for Scoping Reviews) checklist. The protocol was registered with PROSPERO (CRD42024586863).

### Eligibility criteria

Studies were selected based on predefined inclusion and exclusion criteria (Supplement 1). The population of interest comprised children diagnosed with asthma, while studies focusing on adults or mixed populations with inseparable outcomes were excluded. Studies with mixed child-adult populations were excluded unless paediatric-specific remission data could be independently extracted. All types of interventions, including no intervention, were considered, without restriction. The primary outcome was asthma remission, defined as studies that explicitly used the term “remission” or equivalent terminology (resolution, resolved). This inclusive approach was necessary to capture the full scope of definitions used in paediatric asthma literature, given the lack of standardized terminology. Narrative reviews, systematic reviews, and case reports were excluded. Only studies published in English were included.

### Search strategy

We searched for eligible studies in PubMed MEDLINE from January 2010 to February 2024. PubMed MEDLINE was selected as the sole database given its comprehensive coverage of biomedical literature and its suitability for the exploratory aim of this scoping review. The 2010 start date was selected to focus on the most recent decade of literature, capturing contemporary definitions and approaches to paediatric asthma remission while maintaining a manageable scope for this scoping review. This timeframe also establishes a baseline prior to the recent formalization of remission criteria in adult asthma (2020 onwards) and the widespread adoption of biologic therapies in paediatric populations. The search strategy was: ("Asthma"[Mesh] OR asthma[tiab]) AND ("Remission, Spontaneous"[Mesh] OR "Remission Induction"[Mesh] OR remission[tiab] OR resolution[tiab] OR resolved[tiab]).

### Data charting

Data were extracted using a standardized extraction form developed specifically for this review. Two independent reviewers conducted data extraction, and any discrepancies were resolved through discussion and consensus. When necessary, a third reviewer was consulted to solved disagreements (EA, MM, NGP). Key variables included study identification (eg, PMID, first author, country), study design type, sample size, and participant characteristics such as age at inclusion and outcome evaluation. Asthma related information such as asthma severity, definitions of asthma and remission, presence and thresholds of symptoms, medication use, control measures, lung function, and biomarkers (FeNO, eosinophils, AHR) was recorded. Where applicable, time frames for remission and statistical measures used to quantify remission were also documented.

### Collating, summarizing, and reporting the results

Due to variability in study design, definitions of remission, and outcome reporting, a narrative synthesis approach was employed. Findings were grouped based on key characteristics such as specific remission criteria used. Patterns in asthma remission rates and associated factors were identified and summarized. To objectively identify groupings of remission components that could inform the proposed ontology, hierarchical cluster analysis (HCA) was performed. This statistical method was chosen over purely thematic grouping to provide an unbiased, data-driven classification of definition patterns. Specific components of remission definitions were extracted and converted into binary variables (1 = present, 0 = absent). The Jaccard similarity coefficient was used as it is appropriate for binary data and considers only shared presences (1–1 matches), which is suitable for comparing definitions based on included criteria. Ward's linkage method was applied to iteratively merge observations, producing a dendrogram that reveals the similarity structure among studies based on shared definition attributes.

## Results

### Flow diagram of study selection

**Fig. 1:**
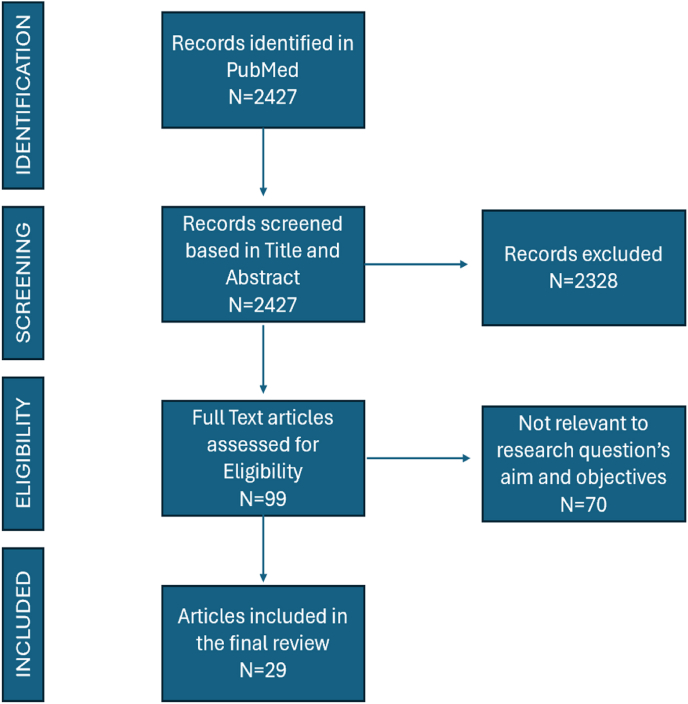
PRISMA-ScR Flow Diagram showing identification, screening, eligibility, and inclusion of studies in the scoping review

A total of 2427 citations were initially identified through the database search. 99 full-text articles were retrieved and assessed for eligibility. 29 studies met the predefined inclusion criteria (see supplement1) and were selected for inclusion in the review. This process is visually represented in the PRISMA-ScR flowchart (Fig. 1).

### Study characteristics

#### Study design

The included studies in this scoping review varied in design, population, and asthma severity. Specifically, there were 13 prospective cohort studies, 12 retrospective cohort studies, and 4 cross-sectional studies.

#### Population

The total number of participants across all studies was 165,895 with sample sizes ranging from 43 to 53893 children. Participants' ages ranged from birth to age 12 years. The studies included both male and female participants, although some studies did not report sex.

#### Countries

These studies were conducted across 26 countries, reflecting a diverse global perspective on asthma remission. The countries included in the studies span multiple continents, such as Europe, North America, and Asia.

#### Asthma definitions and severity

Asthma definition varied across studies; severity was assessed in 8 out of 29 studies while (Supplement 2).

### Remission definitions across studies

#### Definition of remission

The studies in this scoping review defined paediatric asthma remission using various criteria. A summary of the most frequently used criteria in the definition of asthma remission is provided in the Table 1. Detailed definitions of asthma remission are provided in Supplementary Table 3.

**Table 1:** Cross-Tabulation of most frequent criteria defining remission

First Author	No event				No Symptoms			No medication	Normal Lung Function	No BHR
	No Rescue treatment	No Asthma Attack	No Emergency Department visit	No Hospitalization	No Wheezing	No Exercise symptoms	Without any symptoms			
Covar^23^	**+**	**-**	**+**	**+**	**+**	**+**	**+**	**+**	**-**	**-**
Chen^35^	**-**	**-**	**-**	**+**	**-**	**-**	**-**	**+**	**-**	**-**
Hwan Soo^39^	**-**	**+**	**-**	**-**	**-**	**-**	**-**	**+**	**-**	**-**
Zhang^42^	**-**	**-**	**-**	**-**	**+**	**-**	**-**	**+**	**-**	**-**
Hallas^24^	**-**	**-**	**-**	**-**	**-**	**-**	**+**	**+**	**-**	**-**
Carpaij OA^29^	**-**	**+**	**-**	**-**	**+**	**-**	**-**	**+**	**+**	**-**
Longo^9^	**-**	**-**	**+**	**+**	**-**	**-**	**+**	**+**	**-**	**-**
Curry^37^	**-**	**-**	**-**	**-**	**-**	**-**	**+**	**-**	**-**	**-**
Pumputiene^43^	**-**	**-**	**-**	**-**	**-**	**-**	**+**	**-**	**+**	**-**
Xie^44^	**-**	**-**	**-**	**-**	**-**	**-**	**+**	**-**	**+**	**+**
Vonk^38^	**-**	**+**	**-**	**-**	**+**	**-**	**-**	**-**	**+**	**+**
Just^28^	**-**	**-**	**-**	**-**	**+**	**+**	**+**	**+**	**-**	**-**
Marmarinos^45^	**-**	**-**	**-**	**-**	**-**	**-**	**-**	**+**	**-**	**-**
Steinbacher^46^	**+**	**+**	**-**	**-**	**-**	**-**	**-**	**-**	**-**	**-**
Oluwole^32^	**-**	**+**	**+**	**-**	**-**	**-**	**-**	**+**	**-**	**+**
Kim^36^	**-**	**+**	**-**	**-**	**-**	**-**	**-**	**+**	**-**	**-**
Sahiner^30^	**+**	**-**	**-**	**-**	**-**	**-**	**+**	**-**	**-**	**-**
Assar^31^	**+**	**-**	**-**	**-**	**-**	**+**	**+**	**+**	**+**	**-**
Hovland^47^	**-**	**-**	**-**	**-**	**-**	**-**	**+**	**+**	**-**	**-**
Vink^25^	**-**	**-**	**-**	**-**	**-**	**-**	**+**	**+**	**-**	**-**
Javed^48^	**-**	**-**	**+**	**+**	**-**	**-**	**+**	**+**	**-**	**-**
Bobrowska-Korzeniowska^26^	**-**	**-**	**-**	**-**	**-**	**-**	**+**	**+**	**-**	**-**
Tang^34^	**-**	**-**	**-**	**-**	**-**	**-**	**+**	**-**	**-**	**-**
Goldberg^41^	**-**	**-**	**-**	**-**	**-**	**-**	**+**	**+**	**+**	**-**
Owora^33^	**-**	**-**	**-**	**-**	**-**	**-**	**+**	**+**	**-**	**-**
Mogensen^49^	**-**	**-**	**-**	**-**	**+**	**-**	**-**	**+**	**-**	**-**
Just^27^	**-**	**-**	**-**	**-**	**+**	**-**	**-**	**-**	**-**	**-**
Arshad^50^	**-**	**-**	**-**	**-**	**-**	**-**	**+**	**-**	**-**	**-**
Andersson^4^	**-**	**-**	**-**	**-**	**-**	**-**	**+**	**+**	**-**	**-**

Symptom-based Definitions: Most studies (79.3%, 23/29) included the total absence of symptoms in the definition of asthma remission; 3 studies used symptom absence as the sole defining criterion. 55.1% (16/29) of studies referred to the absence of any symptom, while wheezing was specifically mentioned in 24% (7/29). Additionally, exercise-related symptoms included in the definition of only 3/29 (10.3%) studies.

##### Event-based definitions

Acute events were considered as a defining criterion in 41.4% (12/29) of studies. Acute events were identified as need of rescue treatment, asthma attacks/exacerbations, emergency visits or hospitalization. Absence of asthma attacks/exacerbations was specifically included in 20.7% (6/29) studies as a remission-defining criterion. Alternatively, no asthma hospitalization, emergency visits and no use of rescue medication were used separately in the definition in 4/29 (13.8%).

Medication-based definitions: Asthma medications were included in the definition of 68.9% (20/29) of studies. Most however (58.6%, 17/29) did not specify medications.

##### Other parameters

Absence of bronchial hyperresponsiveness (BHR) was used as a criterion in 3/29 (10.3%) studies. School absenteeism was mentioned in 1 study.^23^ Notably, biomarkers such as exhaled nitric oxide (FeNO) or blood eosinophil levels were not included in remission definitions in any of the reviewed studies.

**Fig. 2:**
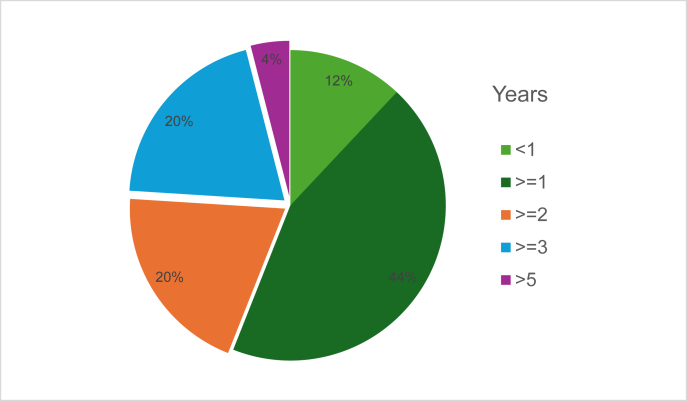
Timeframes for remission diagnosis

###### Timeframe for remission diagnosis (Fig. 2)

The most frequently reported minimum duration was 1 year, used in 11 out of 29 studies (37.9%).^23^, ^24^, ^25^, ^26^, ^27^, ^28^, ^29^, ^30^, ^31^, ^32^, ^33^ Five studies (17.2%) defined remission as being at least 2 years^9^^,^^34^, ^35^, ^36^, ^37^ and 5 studies as at least 3 years (17.2%).^4^^,^^38^, ^39^, ^40^, ^41^ Only 3 studies applied shorter durations of less than 1 year while 1 study as 8 years. In 4 studies the exact timeframe of asthma remission was not defined (Supplement 3). The median time frame across studies was 1 year, while the average time frame was 1.85 years.

##### Cluster analysis

**Fig. 3:**
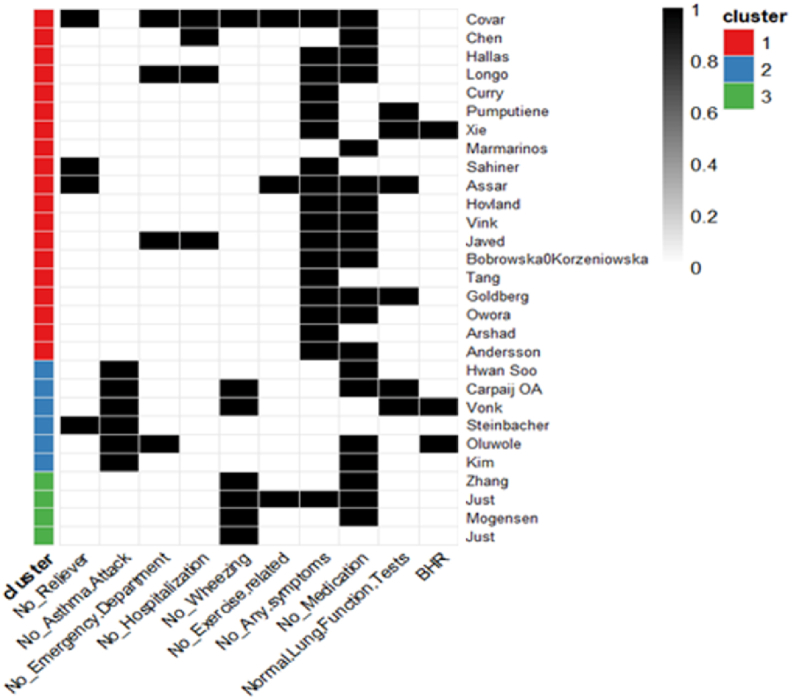
Heatmap clustering according to definitions.

Clustering of the asthma remission definitions based on the criteria used across the 29 studies, identified 3 distinct patterns (Fig. 3). Cluster 1 (red) and Cluster 3 (green) define remission based primarily on the absence of symptoms, with limited inclusion of objective measures or event-based criteria. Cluster 1 refers to any symptoms, while Cluster 3 specifies wheezing and to a less extent exercise-related symptoms. Cluster 2 (blue) emphasize the absence of asthma events, also including objective measures (spirometry, BHR). Use of medication is equally represented among all clusters.

## Discussion

In the last few years, the concept of asthma remission has gained increasing attention and remains a topic of debate. This scoping review reveals how paediatric asthma remission has been historically defined in the literature. While the concept of remission in asthma has been proposed as a therapeutic goal,^13^ especially in the era of precision medicine and biologics, the diversity of criteria significantly limits the comparability of findings across studies, as well as its impact on clinical practice.

A key finding of this review is that most studies have employed clinically based definitions of remission, typically relying on the complete absence of respiratory symptoms. This approach is straightforward to use in clinical practice; however, it may underestimate persistent subclinical disease activity,^51^ particularly in children who report few symptoms but exhibit abnormal lung function or persistent airway inflammation. Furthermore, only a minority of studies incorporated objective measures, such as spirometry or BHR, into their definitions of remission. Notably, no studies included biomarkers such as FeNO or blood eosinophils, despite growing evidence that these markers can reflect underlying airway inflammation, even in asymptomatic individuals. While the reasons for selecting and proposing a definition, depends upon the aims and design of each study, clinical remission generally does not require proof of pathophysiological resolution. In most studies, no symptoms also imply no acute symptoms and hence no attacks, emergency visits or exacerbations. While for research purposes, individual outcomes may be valuable for in-depth analyses, in clinical practice, a simple definition may be preferable. A more stringent definition for “complete remission” could be used as a term could serve to encompass a composite of clinical remission, biologic remission (resolution or suppression of type 2 inflammation, as reflected by biomarkers such as FeNO and eosinophils), and functional remission, characterized by normal or at least stable lung function, particularly relevant for individuals with more severe disease.^10^ Although our review focused on studies published from 2010 onwards, it is important to note that key earlier longitudinal cohorts^38^^,^^52^, ^53^, ^54^, ^55^, ^56^, ^57^, ^58^, ^59^ used remission concepts consistent with our findings, further supporting the robustness of the observed patterns.

Medication-free status, particularly the absence of controller medication, defined remission in close to 70% of studies and probably reflects a tendency to equate remission with a complete resolution of disease. There was inconsistency regarding whether this referred specifically to the absence of inhaled corticosteroids or any anti-asthma medication. Such variation highlights the lack of standardized remission criteria across studies and complicates the comparison of outcomes between different age groups and disease severities. This impacts interpretation, as a child who is symptom-free while on low-dose controller therapy or with mild asthma may have a different disease trajectory than one who is symptom-free without any medication. Nevertheless, the non-negligible remaining 30% of studies already accepted the concept of “on-treatment remission”, without necessarily defining it as such. This shift indicates a gradual move toward recognizing asthma as a chronic, manageable condition rather than one that can only be spontaneously resolved. An approach that clearly distinguishes between “on-treatment remission” without the use of systemic corticosteroids, and “remission without treatment” is increasingly relevant in the current biologic era, particularly in the context of severe asthma^60^ with real-world studies showing that biologics can induce remission in 36.8%–47.4% of children dependent on ACAAI/AAAAI/ATS consensus criteria or International Severe Asthma Registry remission criteria were applied.^19^

The predominance of 1–3-year time frames in defining remission across studies likely reflects a consensus on the need to capture medium-to-long-term disease stability rather than short-term fluctuations more related with asthma control. Asthma, particularly in children, can show intermittent symptom-free periods.^20^ By extending the observation period to more than 1 year, seasonal triggers,^61^ and potential relapses can be accounted for, thereby improving the reliability of remission classification. Only 3 studies, used a time frame of less than 1 year; these have apparently used the term with a different context, ie, distinct phases of disease activity.^43^^,^^44^^,^^46^ In another 4 studies, the exact time frame for defining remission was not clearly specified, which may be attributed to the study design and the scheduling of evaluations across multiple years. While for research purposes, 1 year is an important milestone, for clinical purposes, longer periods may better reflect the natural history of the disease and patient expectations. In recent studies of on-treatment remission in severe adult asthma, extending the remission time frame to 2 years considerably reduced the proportion of subjects achieving this state (varying from 50% to 20%).^62^^,^^63^

Importantly, children who achieve asthma remission may still face an increased risk of accelerated lung function decline in adulthood and the development of persistent airway limitation.^64^ This highlights the importance of long-term monitoring and reinforces that remission is not always complete and not necessarily equal to a permanent cure.

Prior to 2020, remission was commonly defined as the absence of symptoms, based on patient-reported outcomes and treatment history. In 2020, a consensus framework was proposed for adults with severe asthma.^13^ The concept of “clinical remission” was coined, defined as at least 12 months without significant symptoms, stable lung function, no systemic corticosteroid use, and patient agreement. Off-treatment remission included the same criteria with no use of asthma medication. Complete remission required these clinical criteria plus objective evidence of resolved airway inflammation.^13^ In 2022, Thomas et al expanded on these definitions by incorporating remission on-treatment^10^ and 1 year later a consensus from a joint workgroup of the ACAAI, AAAAI, and ATS^16^ proposed 6 criteria for on-treatment remission, all of which must be maintained for at least 12 months:^1^ no exacerbations,^2^ no work or school absences due to asthma,^3^ stable or improved lung function,^4^ use of low-to-medium doses of controller therapy,^5^ ACT score >20, AIRQ score <2, ACQ score <0.75, and^6^ reliever use limited to once per month. In addition to the various definitions of remission, the era of biologics has introduced a distinct group of patients so-called super-responders, who differ from other categories.^60^

Current definitions, criteria, and treatment strategies for asthma remission have been developed considering primarily adult populations, even though remission has been extensively used as a concept in paediatric asthma for a long time. Although comprehensive, the proposed set of adult criteria may not be a perfect fit regarding their applicability to children in clinical practice.^65^ Lung-function–based criteria, such as normal FEV_1_, are difficult to apply in all-age groups, spirometry is often infeasible in children under 5 years old. Airway hyperresponsiveness and inflammation tests (eg, bronchial challenge, FeNO, sputum eosinophils) are rarely used in young children due to technical and feasibility limitations.^18^^,^^65^ Tools like symptom control questionnaires and spirometry are either unsuitable or less accurate for younger children and measures of school attendance or medication adherence may not accurately reflect disease burden or treatment response in children.^66^^,^^67^ In contrast to adults, among whom true disease resolution is rare, a substantial proportion of children experience symptom resolution over time. Our review was not able to determine if the definition of asthma remission is related to child's age as most studies used broad age ranges and remission criteria may differ between preschool children, school-aged children, and adolescents. Thus, predicting which children will achieve remission remains challenging due to the marked heterogeneity in clinical presentation, inflammatory phenotypes, and treatment response.^3^^,^^68^, ^69^, ^70^

### Limitations

As with all scoping reviews, this study did not include a formal quality appraisal of included studies, which limits commentary. Additionally, the review was limited to studies published in English and indexed in PubMed, which may have resulted in the exclusion of some papers.

Although definitions of remission have focused on adult populations with severe asthma, the concept is not exclusive to severe cases and may differ across asthma phenotypes. As highlighted in this review, most included studies did not define the asthma severity of the included population. Different levels of asthma severity may follow distinct trajectories; therefore, this is a weakness of several included studies.

Nevertheless, the evolving definitions of asthma remission are currently more suited to research settings than to routine clinical practice. Their application in everyday paediatric clinical decision-making remains limited. There is a need to clarify paediatric-specific remission definitions that reflect real-world asthma trajectories and support individualized care. To be useful in clinical practice, these definitions must be simple, practical, and easy to use and communicate both between clinicians and between clinicians and patients.

### Conclusion and future directions

Given recent advances in paediatric asthma treatment, including the emerging potential of biologic therapies to achieve remission in children with severe disease, it is timely to revisit the framework used to define and measure this outcome. The historical consensus is that complete absence of clinical symptoms is closer to the idea of remission. Within that scope, both off-treatment and on-treatment remission can be acknowledged. Another future consideration is the ongoing discussion about “disease-modifying treatments”. This concept comes from adult severe asthma, and its relevance to children is still unknown. It also raises the question of when biologic therapy might be stopped in paediatric patients who achieve remission, an area that will require long-term data. Additional studies should compare short and long-term impact of remission definitions. Incorporating biomarkers and closer (eg, digital) monitoring may provide a more precise understanding of disease activity,^14^ and could define children with complete remission that is more likely to represent disease resolution, not necessarily cure.

However, for use in routine clinical practice, definitions of paediatric asthma remission must remain pragmatic. They should capture the real-world variability of paediatric asthma and support clinical decision-making tailored to each child. To ensure both clinical relevance and conceptual clarity, a structured, evidence-informed consensus process is needed to develop a standardized and practical ontology of remission-related concepts tailored to paediatric asthma.

Thus, based on the above findings, on behalf of WAO, a Delphi consensus process has been initiated, to propose clinically relevant and age-appropriate definitions of paediatric asthma remission.

## Authors consent for publication

All authors have given their consent for publication.

## Availability of materials

Not applicable.

## Ethics statement

Each author's potential conflicts of interest have been disclosed.

## Disclosure statement regarding use of generative artificial intelligence (AI) and AI-assisted technologies

Nothing to disclose.

## Funding statement

No funding to report.

## Declaration of competing interest

The authors declare that they have no competing interests.
