# Supplementary material for: The definition of asthma remission in children: A scoping review by the WAO Paediatric Asthma Committee
Source: World Allergy Organ J. 2026 Jan 5;19(1):101166. doi: 10.1016/j.waojou.2025.101166 (PMC12809731; doi:10.1016/j.waojou.2025.101166)
Supplement: Multimedia component 1 [file mmc1.docx]

|  | Included | Excluded |
| --- | --- | --- |
| Population | Children with asthma | Adult population  Mixed populations in which the outcomes are not separable |
| Interventions/Comparators | Any intervention |  |
| Outcomes | Asthma Remission | Diagnosis of remission during adulthood |
| Study Design | Prospective cohort  Retrospective cohort  Cross-sectional  Non-randomized interventional trial  Randomized controlled trial | Narrative Reviews  Systematic Reviews Experimental studies  Case Reports |
| Language | English | Foreign Language Articles |
| Time Period | January 2010- February 2024 |  |

Supplement1. Inclusion and Exclusion Criteria
