# Supplementary material for: The definition of asthma remission in children: A scoping review by the WAO Paediatric Asthma Committee
Source: World Allergy Organ J. 2026 Jan 5;19(1):101166. doi: 10.1016/j.waojou.2025.101166 (PMC12809731; doi:10.1016/j.waojou.2025.101166)
Supplement: Multimedia component 2 [file mmc2.docx]

| First Author  Study Design | Country | Definition of asthma |
| --- | --- | --- |
| Covar et al., Prospective cohort | USA | Asthma activity was defined to occur if any of the following were present during the observational phase: contact with local medical provider, school absence, emergency department visit, and hospitalization due to asthma; presence of wheezing or any other exercise-related symptoms; or use of rescue or controller medications |
| Zhang et al., Prospective cohort | UK | Recurrent wheezing in the last 12 months and either given a clinical diagnostic of asthma with or without treated with asthma medications |
| Hallas et al., Prospective cohort | Denmark | Doctor's diagnosis |
| Xie et al.,  Prospective cohort | China | Guideline for the Diagnosis and Optimal Management of Asthma in Children (2016 Edition) |
| Vonk et al., Prospective cohort | Netherlands | Doctor's diagnosis |
| Just et al.,  Prospective cohort | France | GINA |
| Hovland et al.,  Prospective cohort | Norway | Doctor-diagnosed asthma, asthma symptoms, and the use of anti-asthmatic medication |
| Vink et al.,  Prospective cohort | Dutch | Asthma was defined as a parentally reported physician’s diagnosis of asthma and/or having symptoms of asthma and/or use of asthma treatment prescribed by a physician in the past 12 months |
| Bobrowska-Korzeniowska et al.,  Prospective cohort | Poland | GINA |
| Tang et al.,  Prospective cohort | Hong Kong | American Thoracic Society criteria |
| Just et al.,  Prospective cohort | France | Clinical symptoms (wheeze/asthma symptoms) and physician diagnosis at the follow-up assessment (at six years old) |
| Arshad et al.,  Prospective cohort | UK | Persistent Asthma: Asthma symptoms present at both 10 and 18 years of age.  Adolescent-Onset Asthma: No asthma symptoms at 10 years but present at 18 years. |
| Andersson et al.,  Prospective cohort | Sweden | Use of asthma medication or wheeze during the past 12 months |
| Chen et al.,  Retrospective cohort | Denmark | At least one hospitalisation for asthma or 2 prescriptions for anti-asthmatic medication, less than 12 months apart |
| Kim HS et al.,  Retrospective cohort | Korea | Children who experienced typical asthma symptoms (wheezing, dyspnoea, and chronic cough) within the past 12 months with variable expiratory airflow limitation confirmed by either positive bronchodilator response (BDR) or bronchial hyper-responsiveness (BHR) |
| Carpaij OA et al., Retrospective cohort | Netherlands | Doctor-diagnosed asthma and bronchial hyperresponsiveness (ie, substance provocative concentration causing a 20% drop in FEV1 [PC20] <16 mg/Ml histamine). |
| Longo et al.,  Retrospective cohort | Canada | Canadian criteria Association |
| Steinbacher et al.,  Retrospective cohort | Austria | GINA |
| Kim et al.,  Retrospective cohort | Korea | Doctor's diagnosis according to ATS guidelines |
| Sahiner et al., Retrospective cohort | Turkey | Recurrent wheezing and doctor's diagnosis |
| Assar et al.,  Retrospective cohort | Iran | History of at least two asthma attacks in the past. |
| Javed et al.,  Retrospective cohort | USA | Doctor's diagnosis |
| Goldberg et al., Retrospective cohort | Israel | Doctor's diagnosis |
| Owora et al., Retrospective cohort | Canada | Doctor’s diagnosis plus the presence of asthma symptoms (e.g., wheezing, shortness of breath) and/or asthma medication use within the past 12 months. |
| Mogensen et al.,  Retrospective cohort | Sweden | At least two of the following was reported: wheeze the previous year, asthma medication the previous year or doctor’s asthma diagnosis at any time |
| Curry et al.,  Cross-sectional | USA | Not defined |
| Pumputiene et al.,  Cross-sectional | Lithuania | GINA |
| Marmarinos et al.,  Cross-sectional | Greece | Patients with recurrent wheezing episodes (3 during the previous year), following clinically defined acute ‘‘viral’’ upper respiratory tract infection. |
| Oluwole et al.,  Cross-sectional | Canada | Physician diagnosed asthma with symptoms of any wheezing, asthma medication use, ≥1 asthma episodes, and ≥1 health care services in the past 12 months |

Supplement 2. Asthma Definition GINA: Global Initiative for Asthma, ATS: American Thoracic Society
