# Supplementary material for: The definition of asthma remission in children: A scoping review by the WAO Paediatric Asthma Committee
Source: World Allergy Organ J. 2026 Jan 5;19(1):101166. doi: 10.1016/j.waojou.2025.101166 (PMC12809731; doi:10.1016/j.waojou.2025.101166)
Supplement: Multimedia component 3 [file mmc3.docx]

| First Author | Definition of Remission | Time frame |
| --- | --- | --- |
| Covar et al. | No contact with local medical provider, school absence, emergency department (ED) visit, and hospitalization due to asthma; presence of wheezing or any other exercise-related symptoms; or use of rescue or controller medications | 1 year |
| Zhang et al. | Disease-free.  A participant was determined to have asthma if s/he had experienced recurrent wheezing in the last 12 months and either given a clinical diagnostic of asthma with or without treated with asthma medications. | Not defined |
| Hallas et al. | Absence of symptoms and treatment | 1 year |
| Xie et al. | Disappearance of symptoms and pulmonary function recovery to levels before the acute attack and maintenance of this function for more than three months. | 3 months |
| Vonk et al. | ‘‘Complete remission’’ of asthma was defined as having no current wheeze and no asthma attacks in the previous 3 years, no use of inhaled corticosteroids, normal lung function (FEV1 .90% predicted), and absence of BHR (PC10 .16 mg/ml).  ‘‘Clinical remission’’ was defined as absence of wheeze and asthma attacks and no use of inhaled corticosteroids. | 3 years |
| Just et al. | Absence of wheezing, airway hyperresponsiveness during exercise or night, no use of any anti-asthmatic medication. | 1 year |
| Hovland et al. | Not fulfilling asthma criteria from 10–16 years | Not defined |
| Vink et al. | Having asthma at a specific survey but not at the following survey. | 1 year |
| Bobrowska-Korzeniowska et al. | Absence of symptoms without the use of medications for at least 12 months before the visit | 1 year |
| Tang et al. | Absence of asthma symptoms and use of anti-asthma medications. | 2 years |
| Just et al. | Absence of wheezing episodes. | 1 year |
| Arshad et al. | Asthma symptoms present at 10 years but absent at 18 years. | 8 years |
| Andersson et al. | No use of asthma medication and no wheezing during the past 12 months, as reported at the endpoint and in the two annual surveys preceding the endpoint | >3 years |
| Chen et al. | No hospitalisation or prescription recorded for 2 years. | 2 years |
| Kim HS et al. | No asthma episodes without prescription of asthma medications. Asthma episodes were defined as healthcare utilization with asthma codes (J45 and J46 according to the 10th International Statistical Classification of Diseases and Related Health Problems [ICD-10]) and being prescribed asthma medications. | 3 years |
| Carpaij OA et al. | No wheeze or asthma attacks, having an FEV1/inspiratory vital capacity (IVC) ratio of greater than or equal to 80%, and no use of asthma-related medication. | 1 year |
| Longo et al. | Absence of a hospitalisation or physician visit for asthma or asthma-like symptoms and no dispensed asthma medication | 2 years |
| Steinbacher et al. | No exacerbations or need for rescue medication | 4 months |
| Kim et al. | No asthma attacks and no use of asthma medication, as well as normal MCT results (> 16mg/ml) | 2 years |
| Sahiner et al. | Not experienced any wheezing and had not used a bronchodilator. | 1 year |
| Assar et al. | Clinical remission was defined as the absence of asthma symptoms for at least one year without the use of inhaled corticosteroids and short acting β2 agonists. In the cases with clinical remission, spirometry and exercise tests were done to document complete remission. | 1 year |
| Javed et al. | Absence of (1) asthma symptoms/signs according to clinician's medical record; (2) patient's use of asthma medications; (3) clinic, urgent care, or emergency department (ED) visits for asthma symptoms; and (4) hospitalization for asthma | 3 years |
| Goldberg et al. | No symptoms and no use of asthma treatment.  Asthma was considered resolved only if the physical examination on the day of the evaluation, the spirometry, and the challenge test were normal | 3 years |
| Mogensen et al. | Fulfilling criteria for asthma at 1, 2, 4 and/or 8 years of age, but not at 24 years of age | Not defined |
| Owora et al. | Current asthma state to a no current asthma state, meaning the child no longer met the criteria for asthma (absence of symptoms and medication use). | 1-2 years |
| Curry et al. | Self-reported absence of asthma symptoms | 2 years |
| Pumputiene et al. | Absence of asthmatic symptoms in subjects and near normal PEF and FEV1 (≥80% predicted). | 2 months |
| Marmarinos et al. | Phase was without any treatment. | Not defined |
| Oluwole et al. | Clinical Remission was defined as the absence of active asthma without clinical confirmation (assessed by lung function and BHR tests).  Complete Remission was defined as the absence of active asthma in addition to a negative BHR test (80% pre-ECT). | 1 year |

Supplement table 3. Definition of Remission
